# Risk factor analysis of postoperative pancreatic fistula after distal pancreatectomy, with a focus on pancreas-visceral fat CT value ratio and serrated pancreatic contour

**DOI:** 10.1186/s12893-022-01650-8

**Published:** 2022-06-22

**Authors:** Koki Maeda, Naohisa Kuriyama, Takuya Yuge, Takahiro Ito, Kazuyuki Gyoten, Aoi Hayasaki, Takehiro Fujii, Yusuke Iizawa, Yasuhiro Murata, Akihiro Tanemura, Masashi Kishiwada, Hiroyuki Sakurai, Shugo Mizuno

**Affiliations:** 1 Department of Hepatobiliary Pancreatic and Transplant Surgery, Mie University Graduate School of Medicine, 2-174 Edobashi, Tsu, Mie 514-8507 Japan; 2 Regional Medical Support Center, Mie University Hospital, Tsu, Mie Japan

**Keywords:** Distal pancreatectomy, Postoperative pancreatic fistula, CT value, Serrated pancreas, Visceral fat, Diabetes mellitus

## Abstract

**Background:**

In pancreaticoduodenectomy, the pancreas-visceral fat CT value ratio and serrated pancreatic contour on preoperative CT have been revealed as risk factors for postoperative pancreatic fistulas. We aimed to evaluate whether they could also serve as risk factors for postoperative pancreatic fistulas after distal pancreatectomy.

**Methods:**

A total of 251 patients that underwent distal pancreatectomy at our department from 2006 to 2020 were enrolled for the study. We retrospectively analyzed risk factors for postoperative pancreatic fistulas after distal pancreatectomy using various pre and intraoperative factors, including preoperative CT findings, such as pancreas-visceral fat CT value ratio and serrated pancreatic contour.

**Results:**

The study population included 147 male and 104 female participants (median age, 68 years; median body mass index, 21.4 kg/m^2^), including 64 patients with diabetes mellitus (25.5%). Preoperative CT evaluation showed a serrated pancreatic contour in 80 patients (31.9%), a pancreatic thickness of 9.3 mm (4.0–22.0 mm), pancreatic parenchymal CT value of 41.8 HU (4.3–22.0 HU), and pancreas-visceral fat CT value ratio of − 0.41 (− 4.88 to − 0.04). Postoperative pancreatic fistulas were developed in 34.2% of the patients. Univariate analysis of risk factors for postoperative pancreatic fistulas showed that younger age (P = 0.005), high body mass index (P = 0.001), absence of diabetes mellitus (P = 0.002), high preoperative C-reactive protein level (P = 0.024), pancreatic thickness (P < 0.001), and high pancreatic parenchymal CT value (P = 0.018) were significant risk factors; however, pancreas-visceral fat CT value ratio (P = 0.337) and a serrated pancreatic contour (P = 0.122) did not serve as risk factors. Multivariate analysis showed that high body mass index (P = 0.032), absence of diabetes mellitus (P = 0.001), and pancreatic thickness (P < 0.001) were independent risk factors.

**Conclusion:**

The pancreas-visceral fat CT value ratio and serrated pancreatic contour evaluated using preoperative CT were not risk factors for postoperative pancreatic fistulas after distal pancreatectomy. High body mass index, absence of diabetes mellitus, and pancreatic thickness were independent risk factors, and a close-to-normal pancreas with minimal fat deposition or atrophy is thought to indicate a higher risk of postoperative pancreatic fistulas after distal pancreatectomy.

**Supplementary Information:**

The online version contains supplementary material available at 10.1186/s12893-022-01650-8.

## Background

The postoperative pancreatic fistula (POPF) is the most clinically problematic complication of pancreatectomy. The incidence of POPF depends on the type of pancreatectomy. Distal pancreatectomy (DP), pancreatoduodenectomy (PD) with pancreaticojejunal anastomosis, and PD with pancreatic duct occlusion have reported POFP incidences of 16.0–28.2% [1–5], 10.3–18.5% [6, 7], and 11.8% [8], respectively. Thus, there is a higher incidence of POPF after DP than that after PD [9]. Various risk factors for POPF after DP have been reported thus far, including obesity, younger age, malnutrition, pancreatic thickness, and soft pancreatic texture (i.e., soft pancreas) [10–13]. Although most of these factors can be evaluated preoperatively, pancreatic texture can be determined only by intraoperative findings. Recently, fat deposition in pancreatic parenchyma—related to soft pancreas—has been reported to be strongly associated with POPF after PD, and it is represented by a low pancreatic parenchymal CT value [14, 15]. In addition, preoperative CT images have reportedly been useful in hepatobiliary and pancreatic surgery [16, 17]. In this regard, we have recently reported that the pancreas-visceral fat CT value ratio (PVFR) and serrated pancreatic contour, which can be obtained by preoperative CT images, were associated with fat deposition of the pancreatic parenchyma, and that these factors were selected as strong risk factors of POPF after PD [6]. However, these factors have not been examined for patients that underwent DP.

Thus, the aim of this study was to elucidate the risk factors for POPF after DP and to verify whether PVFR and serrated pancreatic contour could also serve as risk factors for POPF after DP, as they did for POPF after PD.

## Methods

### Patients

Of the 259 patients who underwent DP at the Department of Hepatobiliary Pancreatic and Transplant Surgery of Mie University Hospital with a high volume of pancreatic resection during the 15-year period from January 2006 to December 2020, we excluded three patients whose preoperative CT images could not be referenced and five patients whose pancreatic parenchyma evaluations were difficult (two patients with remnant pancreatic duct stent after pancreaticoduodenectomy, two patients with severe atrophy/calcification, and one patient with pancreatic blastoma). The remaining 251 patients were identified as the study population (Additional file 1: Fig. S1).

The protocol for this research was approved by a suitably constituted Ethics Committee at the institution (Committee of the Institutional Review Board at Mie University of Japan, Approval No. H2021-024), and the study conformed to the provisions of the Declaration of Helsinki. Informed consents were obtained from all the participants through an opt-out form. Participants were explained that they could opt out of participation by filling out an opt-out form. The study received ethical approval for the anonymization of patient data, the absence of risks to the patient, and the potential benefit for the adequate management of POPF based on unbiased information. All data were fully anonymized before we accessed them.

### Risk factor analysis for postoperative pancreatic fistula

Perioperative information and CT images were retrospectively extracted from medical records; univariate and multivariate analyses of risk factors for POPF after DP were performed. Preoperative factors that were evaluated in this study included age, sex, body mass index (BMI), history of preoperative diabetes mellitus (DM), diagnosis, history of preoperative chemoradiotherapy, and the hematologic examinations. Additionally, the preoperative nutritional scores evaluated included the prognostic nutritional index (PNI), neutrophil-to-lymphocyte ratio (NLR), and platelet-to-neutrophil ratio (PNR).

Intraoperative factors that were evaluated included operation time, intraoperative blood loss, surgical procedure (laparotomy or laparoscopy), combined splenectomy, combined portal vein resection, combined celiac axis resection, and simultaneous resection of the alimentary tract. Pancreatic texture could not be confirmed by laparoscopic surgery, and the description of pancreatic texture was omitted in the surgical records in some open surgery cases; therefore, this factor was not evaluated in the present study.

POPF was defined and graded according to the International Study Group on Pancreatic Surgery (ISGPS) in 2016 [18], with Grades B and C set as clinically relevant POPF (CR-POPF). The Clavien-Dindo classification (CD classification) was used for severity classification of postoperative complications [19].

### Evaluation of pancreatic fat deposition and pancreatic morphology on preoperative CT

CT examinations were performed using a multidetector CT scanner; Aquilion ONE (Canon medical systems, Otawara, Tochigi, Japan) or LightSpeed 16VCT (GE Healthcare, Milwaukee, WI, USA) between 2006 and 2011, Aquilion ONE or Discovery 750 HD (GE Healthcare) between 2012 and 2014, Discovery 750 HD or SOMATOM Force (Siemens Healthineers, Erlangen, Bayern, Germany) between 2015 and 2018, and SOMATOM Force or Revolution EVO (GE Healthcare) between 2019 and 2020. After the non-contrast CT scan, a triple-phase contrast-enhanced CT scan was performed with a 1.25-mm slice thickness, including an arterial phase, a portal venous phase, and an equilibrium phase. Contrast material was administered intravenously by a power injector. The arterial phase was obtained using a bolus-tracking technique. The scanning delays of the portal phase were approximately 60–70 s, and those of the equilibrium phase were 130–180 s after the start of the contrast material injection.

**Fig. 1:**
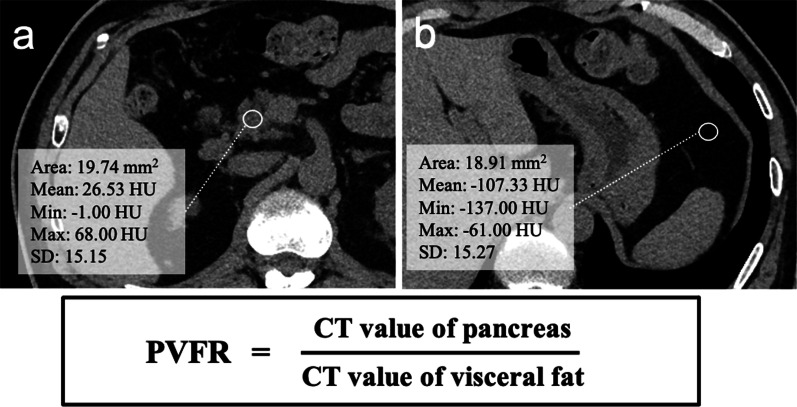
Measurement of the CT value of the remnant pancreatic parenchyma (**a**) and visceral fat (**b**). *HU* Hounsfield units, *PVFR* pancreas-visceral fat ratio

To represent pancreatic fat deposition, the PVFR was determined from preoperative CT by using the method described in our previous report [6]. The CT values were measured at the future remnant pancreatic head for the pancreatic parenchyma and the left side of the stomach for visceral fat (Fig. 1). The CT values (mean values) were measured at four different locations with regions of interest (ROIs) of 15–30 mm^2^ at sites where the vessels did not overlap while comparing the non-contrast CT with the contrast-enhanced CT; the mean CT values of the four measurements was calculated and adopted. The PVFR was calculated as the mean pancreatic parenchymal CT value / mean visceral fat CT value.

**Fig. 2:**
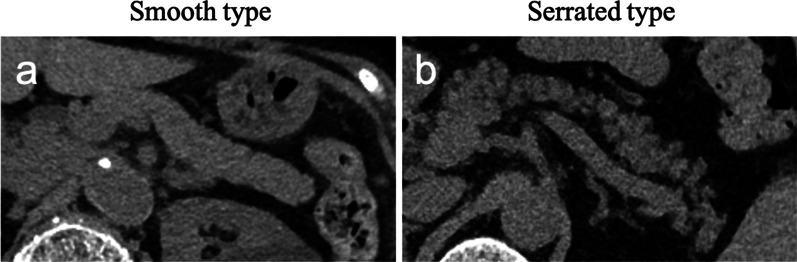
Classification of pancreatic morphology. **a** Smooth type: smooth interlobular border. **b** Serrated type: feathery, irregular interlobular border, and a protrusion of more than 3 mm

Pancreatic morphology was categorized as normal with a smooth margin (smooth type) or an irregular serrated pancreatic contour with protrusions of 3 mm or more (serrated type) (Fig. 2). The pancreatic parenchymal thickness was measured as previously reported [20–22]. It was the value in the direction perpendicular to the long axis of the pancreas on the pancreatic dissection line in the horizontal section of the preoperative CT, identified prior to postoperative CT on postoperative day (POD) 2–17 or according to their operation records.

### Surgical procedure and postoperative management

A total of 34 surgeons performed DP during the study period. Pancreatic stump closure for open surgery was performed using hand-sewn or stapled closure technique. In the case of hand-sewn occlusion of the pancreatic cut end, the pancreatic parenchyma was transected using an ultrasonic coagulation cutting device (SonoSurg; Olympus Optical Co. Ltd., Tokyo, Japan) followed by either main pancreatic duct ligation and suture ligation using the fish-mouth method. Regarding the staple closure technique, pancreatic parenchyma was divided with a bare or mesh-reinforced triple-row stapler (Endo GIA Tri-Staple™ or NEOVEIL Endo GIA Reinforced Reload, Covidien, North Haven, CT, USA) using a purple (3 mm) or black (4 mm) cartridge, selected by the surgeon according to the thickness of the transection line. In laparoscopic surgery, pancreatic stump was closed using a linear stapler or endoscopic hand-sewn technique as well as open surgery. None of the cases involved gastrointestinal anastomosis with the remnant pancreas or pancreatic duct occlusion [23]. A closed suction drain was placed at the pancreatic stump and/or left subphrenic space based on the surgeon’ judgement.

The basic postoperative and POPF management used in this study has been described in our previous report [24]. Prophylactic antibiotics (second-generation cephalosporin) were administered through POD 2. The amylase content of the discharge from the closed-suction drain was evaluated at POD 1, 3, and until drain removal. In the absence of high amylase values more than thrice the upper limit of normal, drains were removed after POD 3. Postoperative CT was routinely performed on POD 6–8 to evaluate peripancreatic fluid collections. Prophylactic somatostatin analogs were not used. A therapeutic antibiotic was immediately initiated when patients showed signs of clinical infection after DP. Regimens of therapeutic antibiotics, which include carbapenem, piperacillin/tazobactam, cephalosporins, ampicillin/sulbactam, and quinolone, were determined by the surgeon empirically or according to the result of bacterial cultures. Postoperative drainage was performed when a peripancreatic fluid collection was detected on postoperative CT and considered to be the source of inflammation.

### Statistical analysis

The normality of data distribution was investigated using the Shapiro–Wilk test; parametric continuous variables were analyzed using Student’s t-test, and the non-parametric continuous variables were analyzed using a Mann–Whitney U test. Categorical variables were analyzed using a χ^2^ test. The significance level was set to P < 0.05; multivariate analysis was conducted using multiple logistic analysis, with variables showing significant differences in univariate analysis set as independent variables. The optimal cut-off value was determined using the Youden index from the receiver operating characteristic (ROC) curve. All statistical analyses were conducted using SPSS (version 26; IBM Japan, Tokyo Japan).

## Results

We retrospectively collected data from 259 patients who underwent DP at the Department of Hepatobiliary Pancreatic and Transplant Surgery of Mie University Hospital from January 2006 to December 2020. The institution is known to have a high volume of pancreatic resections. Three patients were excluded because their preoperative CT images could not be referenced. Another five were excluded due to difficulty in the evaluation of pancreatic parenchyma, and the remaining 251 patients were included in the present study.

### Characteristics of the patients and preoperative CT evaluation

Table 1 presents the data for patient characteristics and intraoperative factors. The median patient age was 68 years (3–89 years), and the study population included 147 male (58.7%) and 104 female (41.3%) patients. The median BMI was 21.4 kg/m^2^ (13.6–34.7 kg/m^2^), and 64 patients had preoperative DM (25.5%); the most common diagnosis was pancreatic ductal adenocarcinoma (PDAC; 105 cases, 41.8%). Preoperative therapy was performed in 67 patients (26.7%): chemoradiotherapy (CRT) was performed in 54 patients and chemotherapy in 13 patients. Thirteen PDAC patients received gemcitabine-based CRT between January 2006 and October 2011. Forty-one patients received S-1 + GEM (GS)-based CRT between November 2011 and December 2020, and the remaining 13 patients received GS chemotherapy. Our preoperative treatment strategies for pancreatic ductal adenocarcinoma have been described in a previous report [25]. The median operation time was 322 min (132–830 min), and the median blood loss was 404 mL (0–5,033 mL). Laparoscopic surgery was performed in 87 patients (34.7%).

**Table 1:** Characteristics of 251 patients that underwent distal pancreatectomy

Characteristics	Value
Age, years	68 (3–89)
Sex, male / female	147 / 104
Body mass index, kg/m^2^	21.4 (13.6–34.7)
Diabetes mellitus, n	64 (25.5%)
Diagnosis, n	
Pancreatic ductal adenocarcinoma	105 (41.8%)
Intraductal papillary mucinous neoplasm	51
Pancreatic neuroendocrine tumor	31
Solid pseudopapillary neoplasm	10
Mucinous cystic neoplasm	9
Metastatic tumor	8
Serous cystic neoplasm	6
Others	31
Preoperative therapy, n	67 (26.7%)
Chemoradiotherapy, n	54
Chemotherapy, n	13
Intraoperative characteristics	
Operation time, min	322 (132–830)
Blood loss, ml	404 (0–5033)
Laparoscopic surgery, n	87 (34.7%)
Without splenectomy, n	25 (10.0%)
Combined PV resection, n	8 (3.2%)
Combined CA resection, n	10 (4.0%)
Stapler closer of pancreatic cut end, n	66 (26.3%)
Simultaneous resection of alimentary tract, n	27 (10.8%)

Data are expressed as number (percentage), median (range), *PV* portal vein, *CA* celiac axis

Preoperative CT evaluation revealed a serrated type pancreas in 80 patients (31.9%) (Table 2). The median pancreatic thickness was 9.3 mm (4.0–22.0 mm), median CT value of the pancreatic parenchyma was 41.8 HU (4.3–73.2 HU), and median PVFR value was -0.41 (-4.88 to -0.04).

**Table 2:** Preoperative CT evaluation before distal pancreatectomy

CT findings	Value
Morphology	
Smooth type	171 (68.1%)
Serrated type	80 (31.9%)
Pancreas thickness, mm	9.3 (4.0–22.0)
CT value	
Pancreas, HU	41.8 (4.3–73.2)
Visceral fat, HU	− 102.1 (− 8.3–-202.2)
PVFR	− 0.41 (− 4.88–-0.04)

Data are expressed as number (percentage), median (range), *HU* Hounsfield units, *PVFR* pancreas-visceral fat CT value ratio

### Incidence and severity of postoperative pancreatic fistula

CR-POPF developed in 86 patients (34.3%), including Grade B in 83 patients (33.1%) and Grade C in three patients (1.2%) (Table 3). Among the patients with Grade B, 30 improved with therapeutic antibiotics alone (CD classification II), while 53 required drain tube replacement and/or additional interventions (CD classification IIIa). Of these 53 patients, 33 (62.3%) required percutaneous drainage. Grade C was observed in three patients, of which one patient underwent additional resection of the residual pancreas at the splenic hilum using the Warshaw technique (CD classification IIIb), one underwent a transverse colon resection and splenic artery stent placement due to puncture of the splenic artery pseudoaneurysm (CD classification IVa), and one developed septic shock and required intensive care in the intensive care unit (CD classification IVb).

**Table 3:** Details of POPF according to Clavien–Dindo classification

POPF	Cases	Clavien–Dindo classification					
		II	IIIa	IIIb	IVa	IVb	V
BL	28 (11.2%)						
CR-POPF	86 (34.2%)						
Grade B	83 (33.1%)	30^a^	53^b^				
Grade C	3 (1.2%)			1^c^	1^d^	1^e^	–

*BL* biochemical leak, *CR-POPF* clinically relevant-postoperative pancreatic fistula

^a^Required antibiotic therapy

^b^Required drain tube replacement and/or additional interventions

^c^Residual pancreas resection at the splenic hilum after Warshaw operation

^d^Transverse colon resection for penetration of splenic artery pseudoaneurysm

^e^Required ICU managements (septic shock)

### Risk factors of clinically relevant postoperative pancreatic fistula

Univariate analysis of the risk factors for CR-POPF identified younger age (P = 0.005), high BMI (P = 0.001), absence of DM (P = 0.002), high preoperative CRP value (P = 0.024), pancreatic thickness (P < 0.001), and high pancreatic parenchymal CT value (P = 0.018) as risk factors (Table 4). However, a serrated type pancreas (P = 0.122) and PVFR (P = 0.373) were not identified as risk factors. The duration of hospital stay was significantly longer in the CR-POPF group (P < 0.001).

**Table 4:** Risk factors of CR-POPF using the univariate and multivariate analyses

Factors	Univariate analysis			Multivariate analysis			
	None POPF / BL (n = 165)	CR-POPF (n = 86)	P-value	β	Odds ratio	CI	P-value
*Preoperative factors*							
Sex, male / female	90 / 75	57 / 29	0.073				
Age, years	70 (3–87)	64 (20–89)	0.005				
BMI, kg/m^2^	21.0 (14.0–30.4)	22.9 (13.6–34.7)	0.001	0.105	1.111	1.009–1.223	0.032
DM, n	52 (31.5%)	12 (14.0%)	0.002	− 1.33	0.265	0.123–0.567	0.001
PDAC, n	75 (45.5%)	30 (34.9%)	0.069				
Preoperative chemoradiotherapy, n	41 (24.8%)	13 (15.1%)	0.075				
*Blood examination*							
White blood count, /μl	5,140 (2,410–12,430)	5,310 (2,530–12,910)	0.35				
Albumin, g/dl	4.1 (2.5–5.0)	4.1 (2.9–5.2)	0.802				
Creatinine, mg/dl	0.7 (0.4–2.6)	0.8 (0.4–1.7)	0.454				
BUN, mg/dl	15.0 (6.0–37.0)	14.0 (5.0–32.0)	0.302				
Cholesterol, mg/dl	187 (103–321)	191 (104–528)	0.135				
Triglyceride, mg/dl	94 (30–328)	109 (34–426)	0.106				
AMY, U/l	75.0 (9.0–1553.0)	74.0 (38.9–275.0)	0.667				
CRP, mg/dl	0.07 (0.01–8.02)	0.11 (0.00–3.38)	0.024				
NLR	2.2 (0.7–12.5)	2.3 (0.8–18.0)	0.978				
PNR	151 (44–667)	153 (54–4,233)	0.996				
PNI	48.0 (30.9–62.1)	48.7 (32.9–60.1)	0.412				
*Intraoperative factors*							
Operation time, min	311 (138–830)	337 (132–754)	0.237				
Blood loss, ml	356 (0–5033)	515 (0–3520)	0.086				
Laparoscopic surgery, n	59 (35.8%)	28 (32.6%)	0.359				
without splenectomy, n	13 (7.9%)	12 (14.0%)	0.098				
Combined PV resection, n	5 (3.0%)	3 (3.5%)	0.556				
Combined CA resection, n	6 (3.6%)	4 (4.7%)	0.467				
Stapler closer of pancreatic cut end, n	47 (28.5%)	19 (22.1%)	0.126				
Simultaneous resection of AT, n	17 (10.3%)	10 (11.6%)	0.451				
*CT evaluation*							
Morphology, smooth / serrated	117 / 48	54 / 32	0.122				
Pancreas thickness, mm	8.8 (4.0–22.0)	10.1 (4.6–20.7)	< 0.001	0.199	1.22	1.092–1.363	< 0.001
CT value							
Pancreas, HU	41.0 (4.5–73.2)	44.2 (4.3–61.8)	0.018				
PVFR	-0.41 (-4.88–-0.05)	-0.42 (-1.19–-0.04)	0.373				
Hospital stays, days	16 (7–99)	36 (7–248)	< 0.001				

Data are expressed as number (percentage), median (range), *CR-POPF*: clinically relevant - postoperative pancreatic fistula, *BL* biochemical leak, *BMI* body mass index, *DM* diabetes mellitus, *PDAC*: pancreatic ductal adenocarcinoma, *BUN* blood urea nitrogen, *AMY* amylase, *CRP* C-reactive protein, *NLR* neutrophil-to-lymphocyte ratio, *PNR* platelet-to-neutrophil ratio, *PNI* prognostic nutritional index, *PV* portal vein, *CA* celiac axis, *AT* alimentary tract, *HU* Hounsfield units, *PVFR* pancreas-visceral fat CT value ratio

**Fig. 3:**
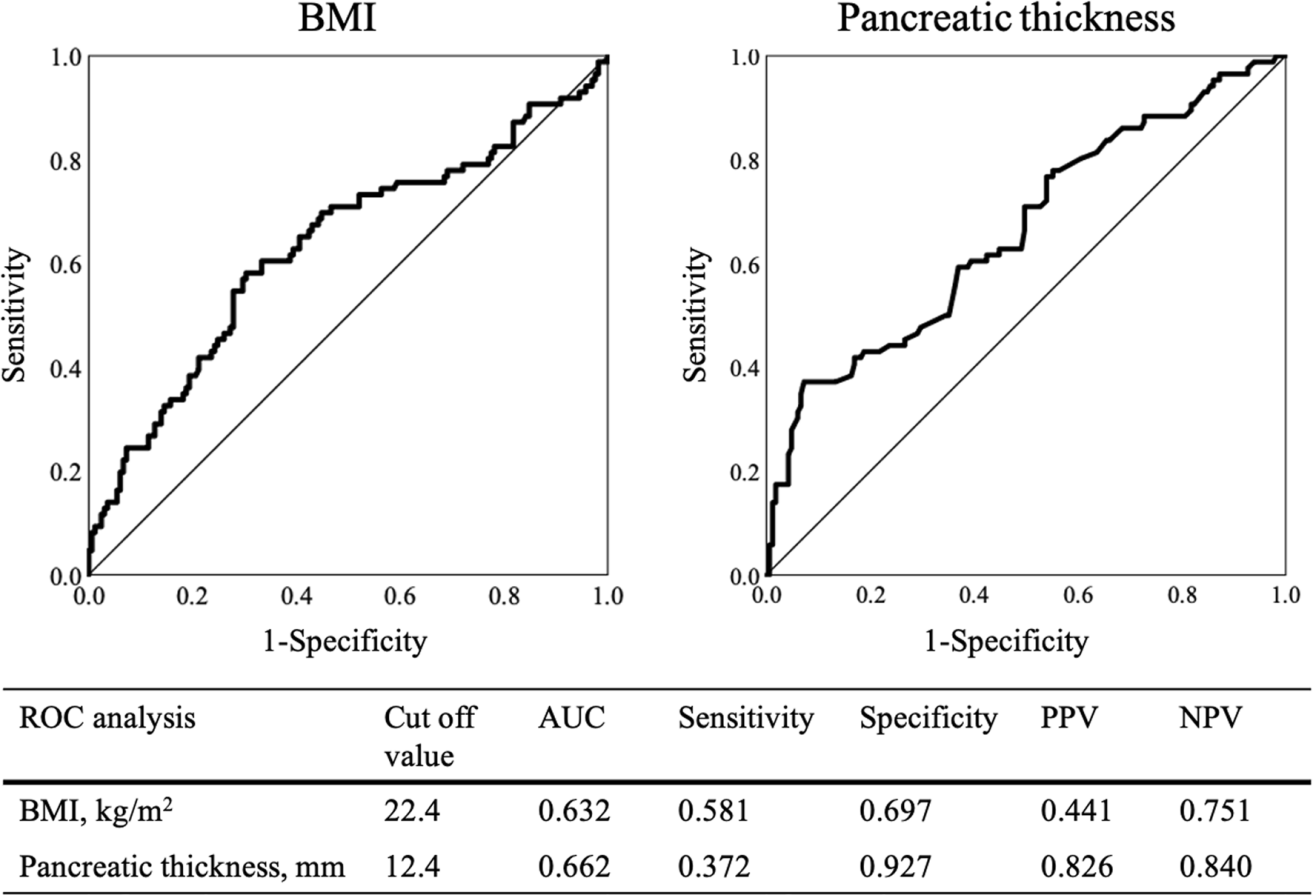
ROC analysis of the risk factors for POPF after DP. *ROC* receiver operating characteristic, *BMI* body mass index, *AUC* area under the curve, *PPV* positive predictive value, *NPV* negative predictive value, *POPF* postoperative pancreatic fistula, *DP* distal pancreatectomy

Multivariate analysis revealed that high BMI (odds ratio: 1.111; 95% CI: 1.709–1.223; P = 0.032), absence of DM (odds ratio: − 1.330; 95% CI: 0.123–0.567; P = 0.001), and pancreatic thickness (odds ratio: 1.220; 95% CI: 1.092–1.363; P < 0.001) were independent risk factors for CR-POPF (Table 4). The optimal cut-off values by ROC analysis were 22.4 kg/m^2^ (AUC: 0.632; sensitivity: 0.581; specificity: 0.697) for BMI and 12.4 mm (AUC: 0.662; sensitivity: 0.372; specificity: 0.927) for pancreatic thickness (Fig. 3).

## Discussion

The present study is the first to show that PVFR and serrated pancreatic contour, which are risk factors for POPF after PD, do not increase the risk of POPF after DP. However, high BMI, absence of DM, and pancreatic thickness were shown to be independent risk factors of POPF after DP.

The incidence of POPF after DP differs between reports, but the results of a large-scale, multi-center, joint study in Japan in 2015 [1] reported an incidence of 28.2% with open surgery and 18.4% with laparoscopic surgery. Several DP-related randomized controlled trials after 2016 [2–4] reported POPF incidence rates of 16.0–18.9%, and a recent meta-analysis [5] reported an incidence of 20.4%, indicating that this complication has a relatively high frequency of occurrence regardless of advancements in surgical techniques. The incidence of CR-POPF in the present study was 34.3%, higher than that in previous reports. In the present study, the incidence of CR-POPF did not differ significantly according to pancreatic stump closure method (hand-sewn closure vs. stapler). Furthermore, the hand-sewn closure method in the laparoscopic surgery—previously adopted in our department—was performed for 33 patients, but CR-POPF was observed in only 10 cases (30.3%); thus, incidence of CR-POPF in our department was not particularly high. Although various reports have evaluated the optimal pancreatic stump closure method for POPF reduction, no consensus has been established, and further studies are needed to identify a closure method that reduces the incidence of CR-POPF. Furthermore, based on our previous report [24], a consideration from the perspectives of intraoperative and postoperative management indicated that the inappropriate drainage tube placement and/or number of drain tubes in our department may have increased the number of cases requiring additional intervention and may have also resulted in excessive use of postoperative therapeutic antibiotics. If the drainage is appropriate, the drain can be removed at an early stage; furthermore, if the indication for therapeutic antibiotic use is judged more strictly, then it would not result in Grade B CR-POPF, and there may be more cases that would heal with a biochemical leak (BL). Although these are all speculations, further improvements are needed in the future both for intraoperative drain tube placement and the postoperative antibiotic usage method.

The previously reported representative risk factors for POPF after DP include obesity, younger age, malnutrition, and soft pancreas [10–13], but many reports have indicated that pancreatic thickness is the most important risk factor [26]. As the global population ages, the demand for pancreatic surgery in elderly patients has increased [27, 28]. Such patients also have a higher incidence of frailty [29], which has been associated with higher morbidity in HBP surgery [30]. Conversely, younger age has been reported as a risk factor for POPF after DP. We consider this as one of the notable features of POPF after DP that differ from other HPB surgery. Univariate analysis in the present study showed that, among the above-mentioned factors, high BMI (obesity), younger age, and pancreatic thickness were detected as risk factors, and multivariate analysis showed that high BMI (obesity) and pancreatic thickness were detected as independent risk factors; these results are similar to those in previous reports. The present study also indicated absence of DM as an independent risk factor in multivariate analysis. Although few report have indicated absence of DM as a risk factor, a recent meta-analysis [5] reported that DM was a significant protective factor for CR-POPF after DP. Exocrine function as well as endocrine function was reduced in DM patients [31], therefore this may have increased the incidence of POPF.

Several reports have performed risk factor analysis of POPF after pancreatic resection by using preoperative CT values, as we have focused on in the present study. In particular, a frequently used value is the pancreas/spleen CT value ratio (P/S ratio), obtained by dividing the pancreatic parenchymal CT value by the spleen CT value; it is based on the liver/spleen CT value ratio commonly used for evaluating fatty liver [32]. The P/S ratio has been reported to represent pancreatic fat deposition and is an index of a soft pancreas in both patients who underwent PD [14] and DP [21], and a significant risk factor of POPF. Furthermore, we had previously discovered that the PVFR, which is based on the P/S ratio and serrated pancreatic contour could be used to predict the fat deposition of the pancreatic parenchyma, and that these findings are risk factors of POPF in PD [6]. Therefore, in the present study, we calculated the PVFR and identified the serrated type pancreas to verify if they could also serve as risk factors of POPF after DP. Specifically, we initially hypothesized that the PVFR and serrated type pancreas, which represent pancreatic fat deposition and a soft pancreas, reflect the fragility of the pancreas, and that a fragile pancreatic stump would be prone to collapse and be more likely to induce POPF. However, our univariate and multivariate analysis showed that the PVFR and serrated type pancreas did not serve as risk factors. Rather, the pancreatic parenchymal CT value was significantly higher in the CR-POPF group, indicating that pancreas fat deposition in this group might be milder than in the non POPF/BL group. Furthermore, an investigation on POPF after DP by Mori et al. [15] indicated that patients with a high pancreatic parenchymal CT value or P/S ratio had mild pancreatic fat deposition and maintained pancreatic exocrine function, and showed a significantly higher POPF incidence than those who do not; these results are similar to the findings of the present study. It is clear that a soft pancreas is a risk factor for POPF after PD, but whether it is a risk factor for POPF after DP, is still controversial [21, 33, 34]. However, the present results seem to indicate that the risk of POPF after DP is higher among patients with less pancreas fat deposition, maintained pancreatic endocrine/exocrine function, less atrophy, and thicker pancreas compared to those with a fragile pancreas with fat deposition. The present results contradict our hypothesis, but to our knowledge, there have been no previous studies that investigated PVFR and morphological characteristics of the pancreas (i.e., serrated type pancreas) as risk factors of POPF after DP; therefore, the present study can provide valuable investigative data in this regard.

The following aspects can be considered as limitations of the present study: (i) this was a retrospective study with a small sample size from a single facility; (ii) the study period was long, potentially introducing various biases related to changes in postoperative management policies, surgical techniques, and pancreatic stump closure method; (iii) the evaluation of pancreatic morphology and CT value measurement method lacked objectivity; although criteria have been set for evaluation of pancreatic morphology, the influence of the evaluator’s subjectivity cannot be excluded; (iv) furthermore, CT value measurements were conducted so as not to overlap with the blood vessels and ducts in comparison with contrast-enhanced CT, but this process was not conducted mechanically, and there were problems with its accuracy. The conclusions drawn from the results of the present study should be interpreted in the light of these limitations and evaluated further in future studies.

## Conclusion

PVFR and serrated pancreatic contour evaluated with preoperative CT did not constitute risk factors of POPF after DP, unlike the findings for PD. High BMI, absence of DM, and pancreatic thickness have been shown to be independent risk factors, and the risk of POPF after DP is thought to be higher among those with a closer-to-normal pancreas with minimal fat deposition or atrophy.

## Supplementary Information

**Additional file 1: Fig. S1.** The number of enrolled cases each year in present study.

## Data Availability

The datasets generated and/or analyzed during the current study are not publicly available as they consist of confidential patient data; however, data will be made available from the corresponding author on reasonable request.

## Abbreviations

BL: Biochemical leak
BMI: Body mass index
CR: Clinically relevant
CRP: C-reactive protein
DM: Diabetes mellitus
DP: Distal pancreatectomy
ISGPS: International Study Group on Pancreatic Surgery
NLR: Neutrophil-to-lymphocyte ratio
PD: Pancreaticoduodenectomy
PNI: Prognostic nutritional index
PNR: Platelet-to-neutrophil ratio
POPF: Postoperative pancreatic fistulas
PVFR: Pancreas-to-visceral fat ratio
ROC: Receiver operating characteristic
ROI: Regions of interest
