# Supplementary figures and images for: Risk factor analysis of postoperative pancreatic fistula after distal pancreatectomy, with a focus on pancreas-visceral fat CT value ratio and serrated pancreatic contour
Source: BMC Surg. 2022 Jun 22;22:240. doi: 10.1186/s12893-022-01650-8 (PMC9215066; doi:10.1186/s12893-022-01650-8)

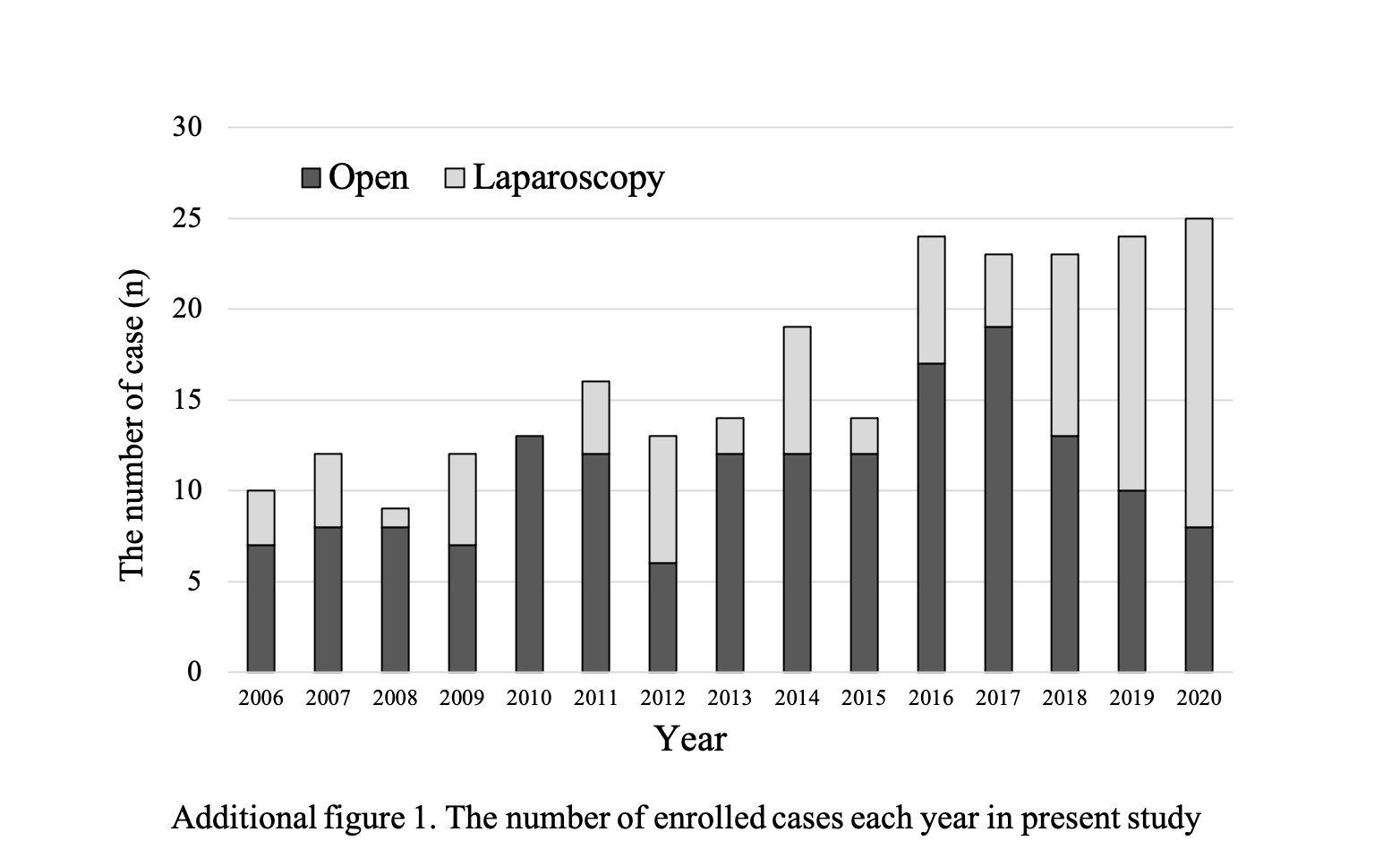

Supplement: Supplementary file 1 — Additional file 1: Fig. S1. The number of enrolled cases each year in present study. [file 12893_2022_1650_MOESM1_ESM.png]
